# Draft Genome Sequences of Three *Shewanella* sp. Strains Isolated from Urban Freshwaters in the Ohio River Valley, USA

**DOI:** 10.1128/MRA.00742-21

**Published:** 2021-09-16

**Authors:** Joy L. Kappesser, Joshua T. Cooper

**Affiliations:** a Department of Biological Sciences, Northern Kentucky University, Highland Heights, Kentucky, USA; University of Southern California

## Abstract

Draft genome sequences of three *Shewanella* sp. strains are reported. The strains NKUCC01_JLK, NKUCC05_KAH, and NKUCC06_TVS were isolated from freshwater sources in the Ohio River Valley, USA. These genome sequences provide insights into *Shewanella* adaptation to urban freshwaters and may help to elucidate their roles in biogeochemical cycling.

## ANNOUNCEMENT

*Shewanella* species are facultative anaerobic, Gram-negative gammaproteobacteria that are primarily aquatic and inhabit diverse environments (1). The diversity of *Shewanella* species is likely underestimated (2), with many uncultivated representatives performing unknown roles in ecological processes. By studying the genomes of *Shewanella* species from urban freshwaters, we hope to evaluate and infer their roles in freshwater biogeochemical cycling.

*Shewanella* sp. strain NKUCC01_JLK and strain NKUCC05_KAH were isolated from subsurface water grab samples collected with a sterile Whirlpak bag from the Licking River, Kentucky, at Frederick’s Landing in January 2021, while strain NKUCC06_TVS was isolated from subsurface water grab samples collected with another sterile Whirlpak bag from Loch Norse on the campus of Northern Kentucky University in January 2021 (coordinates are available in Table 1). Water samples were serially diluted 1:10, spread onto tryptic soy agar (TSA), and grown for 48 h at 25°C. Individual colonies were successively purified on TSA for several months using quadrant streaking. For each strain, genomic DNA was isolated from cells grown in tryptic soy broth at 25°C and was extracted using the UltraClean microbial DNA isolation kit (Qiagen, MD, USA). DNA concentrations were quantified using the Qubit 3.0 broad range kit (Invitrogen) before samples were sent to the Microbial Genome Sequencing Center (MiGS) at the University of Pittsburgh (Pittsburgh, PA). The samples were sequenced to the requested depths using the Illumina NextSeq 2000 platform (2 × 150 bp) to generate paired-end reads. Briefly, libraries were prepared using Illumina Nextera chemistry kits, according to the manufacturer’s protocol, with IDT Illumina 10-bp indices. Reads were examined with FastQC v0.11.5 (3), trimmed with fastp (4), and assembled with Shovill v1.1.0 (5). Assemblies were then annotated with the NCBI Prokaryotic Genome Annotation Pipeline (PGAP) v5.2 (6). Coverage was determined using the read aligner BBMap v38.90 (7) and the trimmed reads. CheckM (8) was used to estimate genome completeness, and data were explored using Kbase v2.3.2 (9). Default software parameters were used throughout unless specified otherwise. Features of the three *Shewanella* sp. strains are summarized in Table 1.

**TABLE 1:** Summary of genome data for the three *Shewanella* sp. strains described in this study

Isolate	Collection site coordinates	Collection site habitat	Assembly size (bp)	G+C content (%)	No. of contigs	*N*_50_ (bp)	Coverage (×)	Size (bp)	Completeness (%)	Contamination (%)	No. of protein-coding sequences	No. of tRNA genes	No. of RNA genes	SRA accession no.	GenBank accession no.	BioSample accession no.
NKUCC01_JLK	39.050611°N, 84.493150°W	Fredericks Landing, shoreline of Licking River, KY	5,109,354	45.21	62	227,063	192	6,688,222	99.68	0.67	4,425	109	129	SRR15168786	JAHKRD000000000	SAMN19515366
NKUCC05_KAH	39.050611°N, 84.493150°W	Fredericks Landing, shoreline of Licking River, KY	5,057,459	45.15	87	176,748	179	6,185,428	99.84	0.41	4,335	100	133	SRR15168782	JAHKQZ000000000	SAMN19515369
NKUCC06_TVS	39 0.031240°N, 84.462720°W	Loch Norse, Northern Kentucky University, KY	5,050,477	45.72	71	223,290	172	5,903,314	99.84	0.37	4,289	99	135	SRR15168780	JAHKQX000000000	SAMN19515371

To explore the taxonomy of our strains, we used Tetra Correlation Search with JSpeciesWS v3.8.2 (10) to select genomes sharing >97% identity. We supplemented the search using PATRIC v3.6.9 Similar Genome Finder (11) and added three closely related genomes from NCBI (Shewanella putrefaciens HRCR-6 [GenBank accession number NZ_JAEU00000000 and RefSeq accession number GCF_000519065.1], Shewanella putrefaciens 97 [GenBank accession number NZ_QNSC00000000 and RefSeq accession number GCF_003315425.1], and *Shewanella* sp. strain WE21 [GenBank accession number NZ_CP023019 and RefSeq accession number GCF_002966515.1]). Pairwise Tetra Correlation analysis indicated that all strains had values of >0.999 with respect to each other and the three additional genomes. Average nucleotide identity on BLAST+ (ANIb) values indicate that all strains and the three additional genomes likely belong to the same species, with ANIb values of >96.7%. Digital DNA-DNA hybridization (dDDH) from the Type (Strain) Genome Server (TYGS) v295 (12) with formula d4 indicated that all three strains shared >70% identity with each other but shared only 40% with any type *Shewanella* species.

In summary, the strains NKUCC01_JLK, NKUCC06_TVS, and NKUCC05_KAH may be representatives of an unnamed clade of freshwater *Shewanella* (with S. putrefaciens HRCR-6, S. putrefaciens 97, and *Shewanella* sp. strain WE21), as illustrated by Thorell et al. (13), which adds to our understanding of freshwater *Shewanella* diversity.

### Data availability.

The whole-genome shotgun projects for *Shewanella* sp. strain NKUCC01_JLK, strain NKUCC05_KAH, and strain NKUCC06_TVS have been deposited in DDBJ/ENA/GenBank under the BioProject accession number PRJNA734631; the versions described in this paper are versions JAHKRD000000000.1, JAHKQZ000000000.1, and JAHKQX000000000.1, respectively. Detailed information can be found in Table 1 regarding BioSample and Sequence Read Archive (SRA) accession numbers. Log files corresponding to the output of the genome assembly by Shovill v1.1.0 and the subsequent graph assembly files for each strain (.gfa) are located at FigShare (https://doi.org/10.6084/m9.figshare.14999802).
